# Clinicopathological Characteristics and Mutation Spectrum of Colorectal Adenocarcinoma With Mucinous Component in a Chinese Cohort: Comparison With Classical Adenocarcinoma

**DOI:** 10.3389/fonc.2020.00917

**Published:** 2020-06-09

**Authors:** Jingci Chen, Liangrui Zhou, Jie Gao, Tao Lu, Jing Wang, Huanwen Wu, Zhiyong Liang

**Affiliations:** Department of Pathology, Molecular Pathology Research Center, Peking Union Medical College Hospital, Chinese Academy of Medical Sciences and Peking Union Medical College, Beijing, China

**Keywords:** AWMC, AC, clinicopathological characteristics, MMR, mutation spectrum, primary site

## Abstract

**Background:** Colorectal adenocarcinoma with mucinous component (AWMC) is a special entity of colorectal cancer. The study is aimed at analyzing the clinicopathological characteristics, mutation spectrum, and prognosis of AWMC and comparing it with classical adenocarcinoma (AC) in a Chinese cohort.

**Methods:** One hundred eight AMWC and 204 AC patients were included. Targeted next-generation sequencing (NGS) was performed on formalin-fixed paraffin-embedded (FFPE) tissues. AWMC was further divided into two groups: AWMC with signet ring cell component and AWMC without signet ring cell component. Clinicopathological features, mismatch repair protein (MMR) status, genetic alterations, and survival outcomes were analyzed after tumor location was taken into consideration.

**Results:** AWMC had larger tumor size (*p* = 0.014) and showed predilection for proximal colon (*p* < 0.001) compared with AC. Regardless of primary sites, AWMC was associated with less metastasis (*p* < 0.001) and earlier AJCC stage (*p* < 0.001). Mismatch repair protein deficiency (dMMR) was more commonly detected in AWMC than in AC for right-sided colon (*p* < 0.001), but the difference was not significant for left-sided colon (*p* = 0.081). The five most commonly mutated genes in AWMC were *KRAS* (45.4%), *TP53* (39.8%), *APC* (22.2%), *PIK3CA* (22.2%), and *SMAD4* (10.2%). AWMC showed a significantly lower mutation rate of *TP53* than AC, both in right-sided colon and in left-sided colon (*p* < 0.001 and *p* = 0.033, respectively). In left-sided colon, AWMC with signet ring cell component had a significantly smaller size than tumors with signet ring cell component (*p* = 0.034). No dMMR cases were detected in AWMC with signet ring cell component (*n* = 7). Moreover, AWMC with signet ring cell component had a significantly lower *KRAS* mutation rate than AWMC without signet ring cell component, both in right-sided colon and in left-sided colon (*p* = 0.036 and *p* = 0.012, respectively). The disease-specific survival (DSS) for AWMC and AC were not statistically different (*p* = 0.0587). Multivariate analysis showed that AWMC was not an independent predictor of prognosis.

**Conclusion:** Regardless of primary sites, AWMC demonstrates less metastasis, earlier stages, more frequent dMMR, and lower *TP53* mutation rate than AC. Our results indicate that different molecular pathogenesis might underlie mucinous morphology in colorectal carcinoma. Mucinous component is not an independent factor of outcome.

## Introduction

Colorectal cancer (CRC) is the fourth most deadly cancer globally and has become a public health problem due to its rising incidence (1). It is characterized by high heterogeneity and varied and outcomes (2). Based on histological subtypes, most CRCs belong to classical adenocarcinomas (AC), with several histological variants associated with specific molecular characteristics (3, 4). Previous research suggested that mucinous histology is related to the proximal colon, microsatellite instability, and numerous upregulated or downregulated genes involved with differentiation and mucin production (5–7). Although the importance of histological appearance has been highlighted, there is a persistent debate regarding its clinical behavior (8–10). It is also unclear whether the existence of signet ring cells is related to clinicopathological characteristics or prognosis (11, 12). In recent years, the location of the tumors has been indicated to be an important predictor factor, which has added to the difficulty of discussing its clinicopathological features and outcomes (13). In this study, we emphasized on colorectal adenocarcinoma with mucinous component (AWMC) after considering the impact of location and assessed the potential differences between AWMC and AC through an institution-based cohort.

## Materials and Methods

### Patients

A total of 312 patients diagnosed with CRC between July 2010 and September 2018 from Peking Union Medical College Hospital (PUMCH), Chinese Academy of Medical Sciences were identified, including 108 AWMC patients and 204 AC patients. Approval for this study was obtained from PUMCH Institutional Review Board. Written informed consents were obtained from all the patients.

### Pathological Evaluations

Tumor sections from formalin-fixed paraffin-embedded (FFPE) samples were stained with hematoxylin-eosin (HE) and reviewed by two experienced pathologists independently. AWMC was defined as adenocarcinoma that shows intra or extracellular mucin secretion. AWMC with signet ring cell component was defined as AWMC with signet ring cells which typically show displacement and molding of the nucleus.

### Clinicopathological Features

Clinicopathological parameters were obtained from medical records and pathological reports. Tumors arising from the cecum, ascending colon, and the right 2/3 of transverse colon were considered to be right-sided and those arising in the left 1/3 of transverse colon, descending colon, sigmoid colon, and rectum were considered to be left-sided. Tumor stage was decided based on the American Joint Committee on Cancer (AJCC) tumor staging system (the eighth edition, 2017).

### Mismatch Repair (MMR) Status

Immunohistochemistry (IHC) for the four MMR proteins (MLH1, PMS2, MSH2 and MSH6) was performed on FFPE slides from more recent specimens, including 33 AWMC cases and 88 AC cases. The antibodies used were: MLH1 (clone ES05, Leica), MSH2 (clone 25D12, Leica), MSH6 (clone PU29, Leica) and PMS2 (clone M0R4G, Leica). Normal colon epithelium and infiltrating lymphocytes were used as internal positive controls. dMMR was defined as complete nuclear loss of expression of one or more of these proteins.

### Target Next-Generation Sequencing (NGS)

DNA of FFPE samples was isolated using a QIAamp DNA FFPE Tissue Kit (Qiagen) according to the manufacturer's protocol. 10 ng of DNA were used as template to generate an amplicon library for sequencing for AC samples. Libraries were prepared using an Ion AmpliSeq Library Kit 2.0 and an Ion AmpliSeq Cancer Hotspot Panel v2 (Life Technologies) and quantified using a Qubit dsDNA HS Assay Kit and a Qubit 2.0 fluorometer (Life Technologies). Sequenceing was performed with an Ion Torrent PGM system (Life Technologies). The readings were mapped to the reference genome (hg19) using the Torrent Mapping Alignment Program. Variants were identified using Torrent Variant Caller (3.6.6; Life Technologies). The SGI OncoAim™DNAPanel (Singlera Genomics, Shanghai, China) was used for preparing DNA libraries of AWMC samples and the Qubit® dsDNA HS Assay kit and a Qubit 3.0 fluorimeter was used for quantification. Each library was quantified with KAPASYBR® FAST universal qPCR Kits. Libraries were pooled in equimolar amount and sequenced with an Illumina Miseq sequencer (Illumina, Hayward, CA, USA). The output data were uploaded for quality control, sequence alignment, and variant calling using a vendor-supplied bioinformatics pipeline.

### Statistical Analysis

Statistical analysis was performed with IBM SPSS statistics Version 23.0 (IBM Corporation, USA). Ages were expressed as means ± s.d. and differences between groups were compared using independent sample *T*-test. χ^2^-Square test or Fisher's exact test was used for nominal scaled variables to compare the clinical features and mutation spectrum. The distribution of ordinal scaled variables (T, N, M, and AJCC stage) was performed by Mann-Whitney *U*-test. Cochran-Mantel-Haenszel (CMH) test was used for analyzing stratified categorical data. Disease-specific survival (DSS) was defined as the interval from the date of treatment to death specifically from colorectal cancer. Log-rank test was used to compare Kaplan–Meier survival curves. The covariated factors with a borderline significance (*p* < 0.2) were included in multivariate analysis, which was performed with a Cox proportional hazards regression model. A *p* < 0.05 was considered to be statistically significant.

## Results

### Clinicopathological Characteristics of AWMC and AC

A total of 108 cases diagnosed with AWMC and 204 cases diagnosed with AC were collected. Their clinicopathological features were shown in Table 1.

**Table 1:** Clinicopathological features of AWMC and AC.

	**AWMC (*n* = 108)**			**AC (*n* = 204) n/%**	**P (AWMC vs. AC)**	**P (AWMC without SRC vs. AC)**
	**Without SRC (*n* = 90) n/%**	**With SRC (*n* = 18) n/%**	***P***			
**Sex**						
Male	55 (61.1)	9 (50.0)	0.381	122 (59.8)	0.926	0.833
Female	35 (38.9)	9 (50.0)		82 (40.2)		
**Age (yr), median**	61 (20-84)	44.5 (25-79)	**0.026**	61 (30-91)	0.084	0.462
**Tumor size (cm)**						
≤ 5	39 (43.3)	13 (72.2)	**0.007**	97 (47.6)	**0.014**	**0.002**
>5	50 (55.6)	3 (16.7)		50 (24.5)		
Unknown	1 (1.1)	2 (11.1)		57 (27.9)		
**Tumor location**						
Right-sided	39 (43.3)	11 (61.1)	0.180	40 (19.6)	**<0.001**	**<0.001**
Left-sided	50 (55.6)	7(38.9)		158 (77.4)		
Multiple	1 (1.1)	0 (0)		5 (2.5)		
Unknown	0 (0)	0 (0)		1 (0.5)		
**T**						
Tis	1 (1.1)	0 (0)	0.488	0 (0)	**0.028**	**0.033**
T1	2 (2.2)	1 (5.6)		2 (1.0)		
T2	12 (13.3)	1 (5.6)		11 (5.4)		
T3	62 (69.0)	13 (72.1)		117 (57.3)		
T4	12 (13.3)	2 (11.1)		32 (15.7)		
Tx	1 (1.1)	1 (5.6)		42 (20.6)		
**N**						
N0	39 (43.4)	6 (33.3)	0.111	44 (21.6)	0.087	**0.026**
N1	29 (32.2)	0 (0)		65 (31.9)		
N2	20 (22.2)	9 (50.0)		46 (22.5)		
Nx	2 (2.2)	3 (16.7)		49 (24.0)		
**M**						
M0	81 (90.0)	15 (83.3)	0.721	88 (43.2)	**<0.001**	**<0.001**
M1	8 (8.9)	2 (11.1)		109 (53.4)		
Mx	1 (1.1)	1 (5.6)		7 (3.4)		
**AJCC Stage**						
I	9 (10.0)	1 (5.6)	0.614	5 (2.5)	**<0.001**	**<0.001**
II	28 (31.1)	5 (27.8)		20 (9.8)		
III	43 (47.8)	8 (44.4)		64 (31.4)		
IV	8 (8.9)	2 (11.1)		109 (53.4)		
Unknown	2 (2.2)	2 (11.1)		6 (2.9)		

*SRC, signet ring cell component. Bold values were statistically significant*.

In both AWMC and AC, patients were predominantly males (59% and 60%). The median ages of AWMC and AC were 60 years (range: 20–84 years) and 61 years (range: 30–91 years), respectively (*p* = 0.084). Compared with AC, AWMC had a significantly larger tumor size (*p* = 0.014), was more frequently right-sided (*p* < 0.001), and presented at an earlier AJCC stage (*p* < 0.001). After excluding cases of AWMC with signet ring cell component (*n* = 18), similarly, AWMC without signet ring cell component was also related to larger tumor size, right-sided colon, and earlier stages compared with AC.

Tumors with signet ring cell component was associated with younger age compared with tumors without signet ring cell component (*p* = 0.026). Tumor size was also shown to be different between the two subgroups (*p* = 0.007): the existence of signet ring cell component was associated with a smaller size, with 72.2% of the tumors no larger than 5 cm. There were no significant differences in gender, tumor location and staging (*p* = 0.381, 0.180, 0.614, respectively).

Since there was obvious distinction in primary tumor sites between AWMC and AC, their clinicopathological characteristics were analyzed after stratification (Table 2, Supplementary Tables 1, 2). Despite the primary sites, AWMC developed less metastasis and presented with an earlier AJCC stage. Nevertheless, other clinical characteristics were not completely the same in subgroups: In right-sided colon, sex, age and tumor size did not differ significantly between AWMC and AC, whereas the existence of signet ring cell component was associated with younger age (*p* = 0.022). In left-sided colon, the median age of AWMC was significantly younger than AC (55 vs. 61, *p* = 0.005). There was no obvious difference in tumor size between AWMC and AC (*p* = 0.103), while AWMC without signet ring cell component showed a larger size than AWMC with signet ring cell component (*p* = 0.034).

**Table 2:** *P*-value comparing clinicopathological features between AWMC without SRC, AWMC with SRC, and AC.

	**Sex**	**Age**	**Tumor size**	**T**	**N**	**M**	**Stage**
**Right-sided**							
AWMC vs. AC	0.703	0.422	0.881	0.145	**0.005**	**<0.001**	**<0.001**
AWMC without SRC vs. AC	0.746	0.895	0.471	0.138	**0.002**	**<0.001**	**<0.001**
AWMC without SRC vs. AWMC with SRC	1.000	**0.022**	0.068	**0.001**	0.371	0.790	0.662
**Left-sided**							
AWMC vs. AC	0.750	**0.005**	0.103	**0.004**	0.537	**<0.001**	**<0.001**
AWMC without SRC vs. AC	0.506	**0.030**	**0.025**	**0.011**	0.978	**<0.001**	**<0.001**
AWMC without SRC vs. AWMC with SRC	0.404	0.164	**0.034**	0.488	0.054	0.426	0.400
**Left-sided (excluding rectal tumors)**							
AWMC vs. AC	0.895	0.059	0.149	0.099	1.000	**<0.001**	**<0.001**
AWMC without SRC vs. AC	0.942	0.266	**0.046**	0.211	0.471	**<0.001**	**<0.001**
**Rectal**							
AWMC vs. AC	0.586	**0.040**	0.381	**0.020**	0.403	**<0.001**	**<0.001**
AWMC without SRC vs. AC	0.405	0.059	0.225	**0.026**	0.501	**<0.001**	**<0.001**

*In left-sided tumors (excluding rectal tumors) and rectal tumors, AWMC without SRC was not compared with AWMC with SRC due to the limited number of cases. Bold values were statistically significant*.

For further details, the rectal tumors were separated from left-sided tumors (Table 2, Supplementary Table 2). In rectum, AWMC occurred at a younger age than AC (*p* = 0.040). In the other sites of the left-sided colon, no difference in sex or age was observed, whereas AWMC without signet ring cell component tended to be larger than AC (*p* = 0.046).

### MMR Status

**Figure 1:**
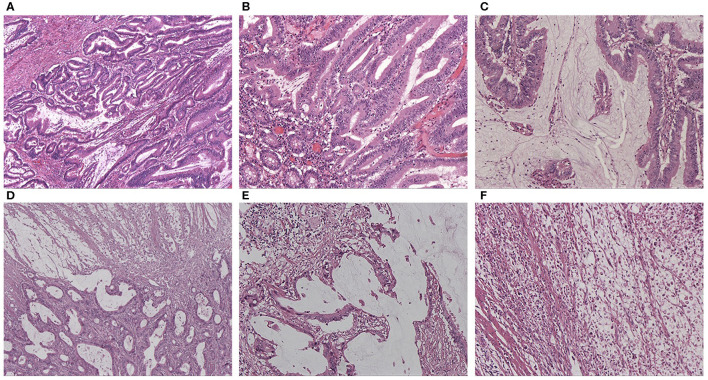
**(A)** HE staining of AWMC showing both classical adenocarcinoma component and mucinous component (HEx40). **(B)** HE staining of AWMC showing classical adenocarcinoma component (HEx100). **(C)** HE staining of AWMC showing mucinous component (HEx100). **(D)** HE staining of AWMC with signet ring cell component (HEx40). **(E)** HE staining of AWMC with signet ring cell component showing mucin pool (HEx100). **(F)** HE staining showing scattered signet ring cells in the same case with D and E (HEx100).

**Figure 2:**
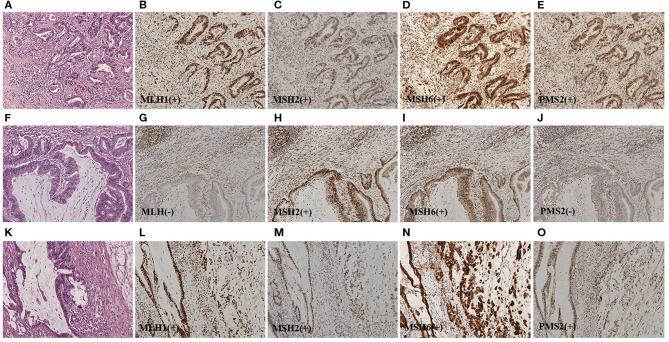
**(A)** Representative HE staining of classical adenocarcinoma (HEx100). For **(B–E)**, representative IHC staining for the four MMR proteins of serial sections from pMMR classical adenocarcinoma (IHCx100). Stromal cells and infiltrating lymphocytes served as internal positive controls. **(F)** Representative HE staining of dMMR AWMC (HEx100). For **(G–J)**, representative IHC staining for the four MMR proteins of serial sections from dMMR AWMC (IHCx100). Stromal cells and infiltrating lymphocytes served as internal positive controls. In this case, MLH1 and PMS2 were negative. **(K)** Representative HE staining of AWMC with signet ring cell component (HEx100). For **(L–O)**, representative IHC staining for the four MMR proteins of serial sections from pMMR AWMC with signet ring cell component (IHCx100). Stromal cells and infiltrating lymphocytes served as internal positive controls.

Deficient MMR (dMMR) was found in 14 of 33 cases (42%) of AWMC and 5 of 88 cases (6%) of AC (Figures 1, 2). AWMC demonstrated a significantly higher frequency of dMMR compared with AC (*p* < 0.001) (Table 3). In dMMR AWMC cases, there were 8 MLH1/PMS2 paired negative, 4 MSH2/MSH6 paired negative, and 2 PMS2 negative. In dMMR AC cases, there were 2 MLH1/PMS2 paired negative, 1 MSH2/MSH6 paired negative, 1 PMS2 negative, and 1 case with all four proteins negative. In AWMC with signet ring cell component, all the 7 tested cases were MMR-proficient (pMMR). Signet ring cell component was associated with less dMMR (*p* = 0.013).

**Table 3:** MMR status and mutation spectrum of AWMC and AC.

	**AWMC**			**AC**	***P***	***P***
	**Without SRC**	**With SRC**	***P***		**(AWMC vs. AC)**	**(AWMC without SRC vs. AC)**
MMR status	n/% (*n* = 26)	n/% (*n* = 7)	**0.013**	n/% (*n* = 88)	**<0.001**	**<0.001**
pMMR	12 (46.2)	7 (100.0)	0.023* (R)	83 (94.3)	0.001* (R)	<0.001* (R)
dMMR	14 (53.8)	0 (0)	1.000* (L)	5 (5.7)	0.081* (L)	0.048* (L)
Mutated gene	n/% (*n* = 90)	n/% (*n* = 18)		n/% (*n* = 204)		
*AKT1*	1 (1.1)	0 (0)	1.000	2 (1.0)	1.000	1.000
*ALK*	0 (0)	1 (5.6)	0.167	0 (0)	0.346	–
*APC*	19 (21.1)	5 (27.8)	0.543	50 (24.5)	0.651	0.526
			0.093* (R)		0.596* (R)	0.260* (R)
			0.662* (L)		0.882* (L)	0.706* (L)
*ATM*	2 (2.2)	2 (11.1)	0.129	1 (0.5)	0.050	0.223
*BRAF*	9 (10.0)	1 (5.6)	1.000	10 (4.9)	0.135	0.101
			1.000* (R)		1.000* (R)	0.737* (R)
			1.000* (L)		0.702* (L)	0.451* (L)
*EGFR*	1 (1.1)	1 (5.6)	0.307	1 (0.5)	0.276	0.519
*ERBB2*	4 (4.4)	1 (5.6)	1.000	1 (0.5)	**0.020**	**0.032**
			1.000* (R)		1.000* (R)	0.494* (R)
			0.417* (L)		0.018* **(L)**	0.044* **(L)**
*FBXW7*	4 (4.4)	0 (0)	1.000	17 (8.3)	0.120	0.233
			–* (R)		0.036* **(R)**	0.116* (R)
			1.000* (L)		1.000* (L)	1.000* (L)
*HRAS*	0 (0)	1 (5.6)	0.167	0 (0)	0.346	-
*KIT*	2 (2.2)	0 (0)	1.000	1 (0.5)	0.276	0.223
*KRAS*	47 (52.2)	2 (11.1)	**0.001**	96 (47.1)	0.776	0.414
			0.036* **(R)**		0.119* (R)	0.436* (R)
			0.012* **(L)**		0.675* (L)	0.234* (L)
*NRAS*	5 (5.6)	2 (11.1)	0.330	8 (3.9)	0.316	0.546
			0.601* (R)		0.127* (R)	0.201* (R)
			–* (L)		0.345* (L)	0.339* (L)
*PIK3CA*	20 (22.2)	4 (22.2)	1.000	28 (13.7)	0.055	0.069
			1.000* (R)		0.263* (R)	0.292* (R)
			1.000* (L)		0.961* (L)	0.807* (L)
*PTEN*	7 (7.8)	1 (5.6)	1.000	7 (3.4)	0.118	0.137
*SMAD4*	9 (10.0)	2 (11.1)	1.000	13 (6.4)	0.229	0.276
			1.000* (R)		0.689* (R)	0.675* (R)
			1.000* (L)		0.264* (L)	0.250* (L)
*SMARCB1*	1 (1.1)	0 (0)	1.000	0 (0)	0.346	0.306
*TP53*	33 (36.7)	10 (55.6)	0.135	135 (66.2)	**<0.001**	**<0.001**
			0.065* (R)		<0.001* **(R)**	<0.001* **(R)**
			0.706* (L)		0.033* **(L)**	0.030* **(L)**

* *P-value after stratified analysis with CMH test. (R), right-sided colon. (L), left-sided colon. Bold values were statistically significant*.

The data were analyzed according to tumors' primary sites. In right-sided colon, AWMC showed more frequent dMMR than AC (*p* < 0.001), and AWMC without signet ring cell component showed more dMMR than AWMC with with signet ring cell component (*p* = 0.023). In left-sided colon, there was no significant difference for MMR status between AWMC and AC (*p* = 0.081). Due to the limited number of cases, only AWMC and AC were compared when classifying left-sided colon into two subgroups (rectum and left-sided colon excluding rectum). There was no statistically difference between AWMC and AC in both groups (data not shown).

### Mutation Spectrum

The five most commonly mutated genes in AWMC were *KRAS* (*n* = 49, 45.4%), *TP53* (*n* = 43, 39.8%), *APC* (*n* = 24, 22.2%), *PIK3CA* (*n* = 24, 22.2%), and *SMAD4* (*n* = 11, 10.2%), while those in AC were *TP53* (*n* = 135, 66.2%), *KRAS* (*n* = 96, 47.1%), *APC* (*n* = 50, 24.5%), *PIK3CA* (*n* = 28, 13.7%), and *FBXW7* (*n* = 17, 8.3%) (Table 3). Compared with AC, AWMC was associated with lower *TP53* mutation rate (39.8 vs. 66.2%, *p* < 0.001) and higher *ERBB2* mutation rate (4.6 vs. 0.5%, *p* = 0.020). The five *ERBB2* mutated spots in AWMC were R678Q (3 cases), V754M, and V842I, and the *ERBB2* mutated spot in AC was V842I. AWMC without signet ring cell component demonstrated similar characteristics to AWMC when compared with AC, with lower *TP53* mutation rate (36.7%) and more frequent *ERBB2* mutation (4.4%). Within AWMC, signet ring cell component was associated with lower *KRAS* mutation rate (*p* = 0.001) (Table 3).

Several frequently mutated genes were selected for analyzing their mutation status in different sites of the colon (Table 3). The mutation spectrum of *APC, BRAF, KRAS, NRAS, PIK3CA, SMAD4*, and *TP53* for AWMC and AC was similar regardless of primary sites. Nonetheless, only in left-sided colon did AWMC demonstrate a higher mutation rate of *ERBB2* than AC (*p* = 0.018). In addition, AWMC showed more frequent mutation of *FBXW7* than AC in right-sided tumors (*p* = 0.036), although their overall mutation rates were not statistically different (*p* = 0.120).

The mutation spectrum of the selected genes was also analyzed in left-sided colon (excluding rectum) and rectum, respectively. There was no obvious difference between AWMC and AC in both sites for mutation rate of *APC, BRAF, FBXW7, KRAS, NRAS, PIK3CA*, and *SMAD4*. For *TP53*, AWMC without SRC demonstrated a lower mutation rate than AC in left-sided colon (excluding rectum) (*p* = 0.042), whereas no obvious difference was observed between AWMC and AC (*p* = 0.083). In rectum, there was no significant difference for *TP53* between AWMC without signet ring cell component, AWMC with signet ring cell component, and AC. For *ERBB2*, all of the mutated cases arose from the left-sided colon (excluding rectum), and the existence of mucinous component was related to a higher mutation rate (AWMC without SRC vs. AC, *p* = 0.042; AWMC vs. AC, *p* = 0.018; AWMC without SRC vs. AWMC with SRC, *p* = 0.495).

***RAS* mutational status**. *KRAS* mutation was detected in 49/108 AWMC patients (45%), whose rate is slightly higher than that of CRC previously reported. Forty-eight of the mutations were point mutation (13 of G12D, 10 of G12V, 9 of G13D, 8 of A146T, 2 of Q61R, 1 of A146V, 1 of G12C, 1 of G12S, 1 of Q61H, 1 of A146T and G13D, 1 of T20M and G13D), with only one insertion (G12_G13insA). Comparison of subgroups indicated that the rate of *KRAS* mutation in AWMC without signet ring cell component was significantly higher than in AWMC with signet ring cell component (52.2 vs. 11.1%, *p* = 0.001) (Table 3). Only one *HRAS* mutation was detected in AMWC. The mutation rates of *NRAS* were low in both groups, with 6.5% in AWMC and 3.9% in AC (*p* = 0.316). Two patients harbored concomitant *NRAS* and *KRAS* mutations, one of whom was *KRAS* (G13D) plus *NRAS* (G12D) and the other was *KRAS* (G12D) plus *NRAS* (G12D).

***BRAF mutational status***. The mutation rates of *BRAF* were 9.3% in AWMC and 4.8% in AC (*p* = 0.135). The hotspot was the typical V600E mutation (7 of V600E, 1 of G466E, 1of G469R, and 1 of R726H).

Several cases with rare concomitant mutations were identified in AWMC without signet ring cell component: one case with *NRAS* and *BRAF* mutation (Q61R and R726H, respectively), one case with concomitant *KRAS* and *BRAF* mutation (A146T and G466E, respectively), and three cases with *BRAF* and *PIK3CA* mutations (E545K and V600E, R108H and V600E, G118D and R726H).

### Correlation of Mutational Status With Clinicopathological Features

The association between *KRAS* or *BRAF* mutation and clinicopathological features was analyzed in AWMC (Table 4, Supplementary Table 3). There was no correlation of sex, age, tumor size, location, or staging with mutation status of *KRAS* or *BRAF*.

**Table 4:** Correlation of *KRAS* and *BRAF* status with clinicopathological features in AWMC.

**Clinicopathological characteristics**	***KRAS*** **status**		***P***	***BRAF*** **status**		***P***
	**Wild (*n* = 59) n/%**	**Mutant (*n* = 49) n/%**		**Wild (*n* = 98) n/%**	**Mutant (*n* = 10) n/%**	
**Sex**						
Male	37 (62.7)	27 (55.1)	0.423	60 (61.2)	4 (40.0)	0.311
Female	22 (37.3)	22 (44.9)		38 (38.8)	6 (60.0)	
**Age (yr), median**	62 (20-84)	60 (25-81)	0.979	58 (20-84)	72 (40-79)	0.070
**Tumor size (cm)**						
≤ 5	31 (52.5)	21 (42.9)	0.278	46 (46.9)	6 (60.0)	0.526
>5	26 (44.1)	27(55.1)		49 (50.0)	4 (40.0)	
Unknown	2 (3.4)	1 (2.0)		3 (3.1)	0 (0)	
**Tumor location**						
Right-sided	27 (45.8)	23 (46.9)	0.968	44 (44.9)	6 (60.0)	0.299
Left-sided	31 (52.5)	26 (53.1)		54 (55.1)	3 (30.0)	
Multiple	1 (1.7)	0 (0)		0 (0)	1 (10.0)	
**T**						
Tis	1 (1.7)	0 (0)	0.635	0 (0)	1 (10.0)	0.535
T1	1 (1.7)	2 (4.1)		2 (2.0)	1 (10.0)	
T2	7 (11.9)	6 (12.2)		12 (12.3)	1 (10.0)	
T3	43 (72.8)	32 (65.4)		70 (71.4)	5 (50.0)	
T4	6 (10.2)	8 (16.3)		12 (12.3)	2 (20.0)	
Tx	1 (1.7)	1 (2.0)		2 (2.0)	0 (0)	
**N**						
N0	24 (40.7)	21 (42.9)	0.254	40 (40.8)	5 (50.0)	0.492
N1	10 (16.9)	19 (38.8)		27 (27.6)	2 (20.0)	
N2	21(35.6)	8 (16.3)		27 (27.6)	2 (20.0)	
Nx	4 (6.8)	1 (2.0)		4 (4.0)	1 (10.0)	
**M**						
M0	54 (91.5)	42 (85.7)	0.328	86 (87.8)	10 (100.0)	0.286
M1	4 (6.8)	6 (12.3)		10 (10.2)	0 (0)	
Mx	1 (1.7)	1 (2.0)		2 (2.0)	0 (0)	
**Stage**						
I	5 (8.5)	5 (10.2)	0.623	9 (9.2)	1 (10.0)	0.298
II	19 (32.2)	14 (28.6)		29 (29.6)	4 (40.0)	
III	28 (47.4)	23 (46.9)		47 (47.9)	4 (40.0)	
IV	4 (6.8)	6 (12.3)		10 (10.2)	0 (0)	
Unknown	3 (5.1)	1 (2.0)		3 (3.1)	1 (10.0)	

### Survival Analysis

**Figure 3:**
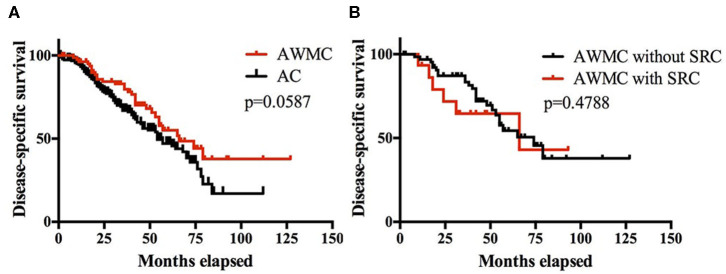
**(A)** Kaplan-Meier survival curves showing DSS of patients of AWMC and AC. **(B)** Kaplan-Meier survival curves showing DSS of patients of AWMC with SRC and AWMC without SRC.

A total of 269 patients (86%) had clinical follow-up, including 188 AC, 66 AWMC without signet ring cell component, and 15 AWMC with signet ring cell component. The median follow-up time was 36 months (range: 1–127 months). The median DSS for AWMC and AC was 66 months and 54 months, respectively. The cumulative survival rates for AWMC at 1, 3, and 5 years were 96, 80, and 55%, respectively. The cumulative OS rates for AC at 1, 3, and 5 years were 93, 67, and 47%, respectively. The DSS of AWMC was not statistically different from AC (*p* = 0.0587) (Figure 3A). Within AWMC, the median DSS was 74 months for patients without signet ring cell component, and 66 months for patients with signet ring cell component. There was no difference between the two subgroups (*p* = 0.4788) (Figure 3B).

To further assess the potential impact of mucinous component on survival, univariate and multivariate analysis of CRC was performed. In univariate analysis, absence of lymph node metastasis (*p* = 0.037) and an earlier AJCC stage (*p* = 0.006) presented a favorable impact on survival (Table 5). Age, histopathology, lymph node status, and stage were included into multivariate analysis. Age over 55 years (HR 1.84, 95% CI 1.12–3.04) and AJCC stage III-IV (HR 1.82, 95% CI 1.08–3.06) were found to be independent predictors of poor outcome (*p* = 0.017 and *p* = 0.025). Nevertheless, histopathology or lymph node metastasis was not a significant predictor of survival (*p* = 0.446 and *p* = 0.543).

**Table 5:** Univariate analysis of DSS of CRC patients.

**Variables**	**HR**	**95% CI**	***P***
**Sex**			
Male	1		0.884
Female	1.03	0.71–1.49	
**Age (yr)**			
≤ 55	1		0.054
>55	1.50	1.00–2.12	
**Tumor size**			
≤ 5	1		0.796
>5	0.95	0.62–1.44	
**Tumor location**			
Right-sided	1		0.782
Left-sided	0.94	0.62–1.43	
**Histopathology**			
AWMC	1		0.059
AC	1.48	0.99–2.14	
**T**			
T1-T2	1		0.211
T3-T4	1.5	0.82–2.5	
**Lymph node metastasis**			
No	1		**0.037**
Yes	1.61	1.03–2.42	
**AJCC stage**			
I-II	1		**0.006**
III-IV	1.99	1.19–2.74	
***KRAS*** **status**			
Wild	1		0.496
Mutant	1.13	0.79–1.65	
***BRAF*** **status**			
Wild	1		0.242
Mutant	1.49	0.73–3.66	

*HR, hazard ratio. CI, confidence interval. Bold values were statistically significant*.

## Discussion

Our study identifies the clinicopathological features, molecular spectrum, and clinical outcomes of AWMC and compare with AC to reveal the potential differences underlie different histology.

Previous studies suggest that special histology subtypes of CRC differ from AC in tumor biology and outcomes (10). Mucinous adenocarcinoma present homologous clinicopathological manifestations such as affecting younger age, arising from proximal colon, and larger tumor size, and signet ring cells indicate poorer outcome (11, 14, 15). However, their clinical features, especially survival outcomes, have been largely controversial (16). In recent years, more researchers have been focusing on the specific impact of site of origin in the pathogenesis of CRC (16–19). In a study of 2,413 colorectal tumors in 2017, Salem et al. reported that colorectal tumors with different primary sites present with distinct clinical features and molecular features (13). Similar conclusions have been illustrated in other studies (18, 20). The findings regarding variations between right-sided and left-sided colon should certainly be considered when exploring the potential differences between tumors with specific histology and AC. In addition, although it is common to include rectum into left-sided colon, some researchers have separated them and found variations (13, 16, 21–23). Nonetheless, this confounding variable was not considered in most of the previous studies emphasizing on histology (11, 24). Therefore, one of the strengths of our research is to compare AWMC with AC after clear stratification.

Firstly, our retrospective study illustrates that AWMC is associated with larger tumor size and proximal colon. Contrary to previous studies, AWMC is more diagnosed at T2-T3 stage, whereas AC demonstrates more metastasis, probably due to earlier symptoms of AWMC (25). We do not find significant difference of gender, tumor location, or staging between AMWC with signet ring cell component and AWMC without signet ring cell component, which is consistent with previous research (8). Nevertheless, AWMC with signet ring cell component appears to occur in younger patients and the tumor size tends to be smaller at diagnosis. Next, AWMC is compared with AC after classifying tumor location into left-sided and right-sided. We find that AWMC is always associated with less metastasis and earlier stage regardless of primary site, which highlights the influence of specific mucinous histology. There is not significant variation of metastatic status or AJCC stage among the two subgroups of AWMC. Interestingly, only in left-sided colon did AWMC show a younger age than AC. After further classifying left-sided colon, we find that the different age distribution is seemingly caused by rectal AWMC rather than other sites of left-sided colon.

In MMR IHC analysis, our results indicate that AWMC is associated with dMMR (*p* < 0.001 in all cases; *p* = 0.001 in right-sided cases). In left-sided cases, the MMR status between AWMC and AC is not significantly different. However, due to the limitation of study population (12 cases arising from left-sided colon), there is still a trend for AWMC to present more dMMR (*p* = 0.081). Notably, none of the AWMC with signet ring cell component (7 cases) were dMMR, which to some extent reveals its unique molecular pathogenesis.

The pathogenesis of CRC involves a series of genetic and epigenetic modifications regulating cell proliferation, apoptosis, and angiogenesis (26). *RAS* and *BRAF* are two well-known proto-oncogenes located downstream of the epidermal growth factor receptor (EGFR) signaling cascade and their mutation results in constitutive activation of EGFR pathway and colorectal tumorigenesis. Mutations of multiple genes such as *KRAS* and *NRAS* confer resistance to anti-EGFR therapy. *BRAF* gene encodes a protein which is part of the *Ras-Raf-MEK-ERK* (MAPK) signaling pathway. *BRAF* mutation is associated with poor prognosis, and is more frequent in signet ring cell carcinoma than AC (27). The mutation rates of *KRAS* and *BRAF* in overall CRC in different ethnic groups and different studies vary a lot, and hotspot genes are important for deciding treatments and predicting outcomes (28). However, the mutational status of CRC in Chinese population is relatively lacking. One of the largest Chinese cohort collecting 1,110 patients has illustrated the molecular spectrum of CRC, but the histology subtypes were not stratified (29). Our data for both *KRAS* and *BRAF* were similar to previously reported rates in mucinous adenocarcinoma, and further stratification indicates similar characteristics of AWMC and AC in each specific location (27).

Previous research suggests that *TP53* is the most commonly mutated gene in CRC. In our study, AC has frequent *TP53* mutation (66.2%), whereas a significantly lower *TP53* mutation rate in AWMC is observed (39.8%, *p* < 0.001). Instead, *KRAS* is the most commonly mutated gene in AWMC (45.4%). The majority of *KRAS* mutations occur in codon 12 or 13, with G12D the most common, followed by the G12V, G13D, and A146T. These data differ slightly from that of western populations and oriental AC patients, suggesting that race and histological subtype might play a role in mutation patterns. Notably, AWMC shows distinct mutation rate of *ERBB2* from AC (4.6 vs. 0.5%, *p* = 0.020), especially in left-sided colon (*p* = 0.018). The mutation spots in 4 AWMC cases were c.2033G>A (p.R678Q) (2 cases), c.2260G>A (p.V754M), and c.2524G>A (p.V842I), respectively. According to previous literature, the functional effect of R678Q is controversial; V754M has never been reported; V842M has been proved to be a functioning mutation in other carcinomas (30, 31). Within AWMC, tumors with signet ring cell component has a significantly lower *KRAS* mutation rate than tumors without signet ring cell component despite the site of origin, which is consistent with our expectations and previous reports (32). In summary, the mutation status of genes of major concern (*APC, BRAF, KRAS*, and *TP53*) do not seem to be influenced by primary sites, and AWMC shows distinct mutation spectrum from AC, mainly attributed to their histology. *ERBB2* and *FBXW7* are two genes with different mutation pattern in different sites, however, more investigation is needed considering the small number of cases in our cohort.

We also notice that despite the low mutation rates of *HRAS* (0.3%) and *NRAS* (4.8%) in CRC overall, AWMC with signet ring cell component has more frequent *HRAS* and *NRAS* mutations than AWMC without signet ring cell component. The trend is likely masked by the small sample size, but it indicates that the existence of signet ring cell component is the marker of specific molecular changes.

Mutation rates of *BRAF* are reported to be higher in signet ring cell carcinoma than in AC (32, 33). Interestingly, we detect only one *BRAF* mutation in 18 cases of AMWC with signet ring cell component, likely due to the ethnics and relative small sample size.

*RAS* and *BRAF* mutations are traditionally thought to be nearly mutually exclusive (34). Concomitant *KRAS* and *BRAF* mutant CRCs are extremely rare (0.001%) are often associated with more advanced stage, therefore it is recommended that *KRAS*-mutated patients not be tested for *BRAF* mutation (35). Nonetheless, recent cases indicate that the occurrence of concomitant *KRAS* and *BRAF* mutations in surgical cohorts maybe higher than hypothesized, and present variable survival outcomes (35). It still needs studying which detailed gene profiling pattern of concomitant mutated tumors is, and which tumor gene signature it imitates more. In our study, the case where both *KRAS* and *BRAF* mutations are identified is a 75-year-old female with a 3 cm tumor in rectum. Both mutations are in the less frequent sites (A146T in *KRAS* and G466E in *BRAF*) and this combination is completely new. Her tumor staging is T1N0M0. However, she was found to have vagina metastases 1 year after the rectal surgery (Miles) and was given radiotherapy both prior to and after a second resection in vagina. It is notable since most previous studies regarding *KRAS/BRAF* concomitant mutations involve *KRAS* codon 12 or codon 13 and *BRAF* V600E, and there is a heterogeneity in the biological features of these mutations (34–38). Some cases show that concomitant *KRAS/BRAF* mutant patients had poorer prognosis (36). Thus, such cases should be kept in mind to clarify the type of concomitant mutations and elucidate their biological behavior.

*KRAS* and *BRAF* mutated CRCs are associated with distinct clinicopathological features according to a large cohort of CRC in Western entity, where *RAS* mutation is associated with male gender and classical adenocarcinoma subtype (39). In Chinese group, previous studies mention that *KRAS*-mutated or *BRAF*-mutated CRCs tend to occur in the proximal colon but have no specific trend in sex, age, lymph node metastasis, or TNM stages (29). Our study focuses on AWMC and AWMC without signet ring cell component and found no distinct features between *KRAS*-mutant and wild-type patients. The prognostic value of *KRAS* and *BRAF* in AWMC requires more research. Due to the small number of cases, we do not manage to compare *KRAS*- or *BRAF*- mutated cases with wild-type cases in AWMC with signet ring cell component.

A set of other somatic mutations is summarized in previous reports but never completely evaluated in Chinese group (27, 28). Since the response of wild-type CRC to anti-EGFR therapy is not as effective as expected, it is important to identify other potential markers to evaluate the outcome. Our research includes mutational profiling of those hotspot mutations as listed in Table 3 (partially). Among the 16 *PIK3CA* mutant AWMC cases, 11 are accompanied with *KRAS* mutations, while there is no significant correlation between *KRAS* and *PIK3CA* mutation (*p* = 0.145), which is inconsistent with previous studies in CRCs and suggests the difference in molecular spectrum between AWMC and other types of CRC (29). Four cases of AWMC with signet ring cell component are found to harbor *PIK3CA* mutations, and one of them has concomitant *KRAS* mutation.

Mucinous histology is reported to be an independent adverse prognostic predictor in some studies, but not in others (11, 40). In our study, AWMC tends to have a favorable prognosis compared with AC, but not significantly. Univariate analysis of DSS indicates that an earlier stage and absence of lymph node metastasis present a better outcome. Multivariate analysis reveals that age and stage are independent prognostic factors. We infer that mucinous histology itself may not influence the survival outcome, but the close association between AWMC and earlier stages are having an impact on prognosis. There is no difference in DSS between AWMC with signet ring cell component and AWMC without signet ring cell component. However, considering the relatively small population, especially for AWMC with signet ring cell component, larger and multi-center studies might be a further direction.

Several limitations are of concern in our study. First, the data are gathered from a single center, and some is incomplete. Second, the majority of the patients coming to our hospital are in earlier stages, which might bring bias when analyzing the relationship between molecular profiling and biological behavior. Third, the population is relatively small with loss to follow up. Nevertheless, our research manages to focus on histology after taking primary sites into consideration and highlights the clinical significance of the unique entity of AWMC. We emphasize that this topic requires more exploration in fields of molecular biology, which will certainly provide clues for treatment. Our study also opens up avenues for larger, multi-center studies with more follow up.

## Conclusion

Regardless of primary sites, AWMC demonstrates less metastasis, earlier stages, more frequent dMMR, and lower *TP53* mutation rate compared with AC. Within AWMC, AWMC with signet ring cell component is associated with lower *KRAS* mutation rate. Mucinous histology does not have an obvious effect in DSS of CRC. Our results indicate the unique molecular pathogenesis underlying AWMC.

## Data Availability Statement

The raw data of the article will be made available by the authors, without undue reservation, to any qualified researcher.

## Ethics Statement

The institutional review board of Peking Union Medical College Hospital approved the study. Written informed consent was obtained from all patients.

## Author Contributions

JC, LZ, and HW collected the cases, analyzed the data, and wrote the manuscript. JG, TL, and JW performed the NGS sequencing and analyzed the results. HW and ZL contributed to the concept and design for the study. All authors contributed to critical interpretation of data and the final draft of the manuscript.

## Conflict of Interest

The authors declare that the research was conducted in the absence of any commercial or financial relationships that could be construed as a potential conflict of interest.
