# Supplementary material for: Clinicopathological Characteristics and Mutation Spectrum of Colorectal Adenocarcinoma With Mucinous Component in a Chinese Cohort: Comparison With Classical Adenocarcinoma
Source: Front Oncol. 2020 Jun 9;10:917. doi: 10.3389/fonc.2020.00917 (PMC7296099; doi:10.3389/fonc.2020.00917)
Supplement: Supplementary file 1 [file Table_1.docx]

Supplementary Table 1. Clinicopathological features of AWMC and AC in right-sided and left-sided colon

|  | Right-sided | | |  | Left-sided | | | | | |
| --- | --- | --- | --- | --- | --- | --- | --- | --- | --- | --- |
|  | AWMC (n=50) | | AC  (n=40)  n/% |  | AWMC (n=57) | | | | AC  (n=158)  n/% | |
|  | Without SRC  (n=39)  n/% | With SRC  (n=11)  n/% |  |  | Without SRC  (n=50)  n/% | | With SRC  (n=7)  n/% | |  |  |
| Sex |  |  |  |  | |  | |  | |  |
| Male | 22 (56) | 6 (55) | 24 (60) | 33 (66) | | 3 (43) | | 96 (61) | |  |
| Female | 17 (44) | 5 (45) | 16 (40) | 17 (34) | | 4 (57) | | 62 (39) | |  |
| Age (yr), median | 65.5 (35-84) | 56 (25-79) | 64 (34-81) | 57 (20-81) | | 41 (28-71) | | 61 (31-91) | |  |
| Tumor size (cm) |  |  |  |  | |  | |  | |  |
| ≤5 | 12 (31) | 6 (55) | 12 (30) | 27 (54) | | 7 (100) | | 80 (50) | |  |
| >5 | 26 (66) | 3 (27) | 18 (45) | 23 (46) | | 0 (0) | | 31 (20) | |  |
| Unknown | 1 (3) | 2 (18) | 10 (25) | 0 (0) | | 0 (0) | | 47 (30) | |  |
| T |  |  |  |  | |  | |  | |  |
| Tis | 1 (3) | 0 (0) | 0 (0) | 0 (0) | | 0 (0) | | 0 (0) | |  |
| T1 | 0 (0) | 0 (0) | 0 (0) | 2 (4) | | 1 (14) | | 2 (1) | |  |
| T2 | 3 (8) | 0 (0) | 0 (0) | 9 (18) | | 1 (14) | | 11 (7) | |  |
| T3 | 26 (66) | 8 (73) | 23 (57.5) | 36 (72) | | 5 (72) | | 89 (57) | |  |
| T4 | 8 (20) | 2 (18) | 10 (25) | 3 (6) | | 0 (0) | | 21 (13) | |  |
| Tx | 1 (3) | 1 (9) | 7 (17.5) | 0 (0) | | 0 (0) | | 35 (22) | |  |
| N |  |  |  |  | |  | |  | |  |
| N0 | 22 (57) | 5 (46) | 8 (20) | 17 (34) | | 1 (14) | | 36 (23) | |  |
| N1 | 11 (28) | 0 (0) | 9 (22.5) | 17 (34) | | 0 (0) | | 51 (32) | |  |
| N2 | 4 (10) | 4 (36) | 12 (30) | 16 (32) | | 5 (72) | | 33 (21) | |  |
| Nx | 2 (5) | 2 (18) | 11 (27.5) | 0 (0) | | 1 (14) | | 38 (24) | |  |
| M |  |  |  |  | |  | |  | |  |
| M0 | 33 (84) | 9 (82) | 18 (45) | 47 (94) | | 6 (86) | | 68 (43) | |  |
| M1 | 5 (13) | 1 (9) | 20 (50) | 3 (6) | | 1 (14) | | 85 (54) | |  |
| Mx | 1 (3) | 1 (9) | 2 (5) | 0 (0) | | 0 (0) | | 5 (3) | |  |
| AJCC Stage |  |  |  |  | |  | |  | |  |
| I | 3 (8) | 0 (0) | 0 (0) | 6 (12) | | 1 (14) | | 5 (3) | |  |
| II | 18 (46) | 5 (46) | 6 (15) | 10 (20) | | 0 (0) | | 14 (9) | |  |
| III | 11 (28) | 4 (36) | 13 (32.5) | 31 (62) | | 4 (58) | | 49 (31) | |  |
| IV | 5 (13) | 1 (9) | 20 (50) | 3 (6) | | 1 (14) | | 85 (54) | |  |
| Unknown | 2 (5) | 1 (9) | 1 (2.5) | 0 (0) | | 1 (14) | | 5 (3) | |  |
