# Supplementary material for: Clinicopathological Characteristics and Mutation Spectrum of Colorectal Adenocarcinoma With Mucinous Component in a Chinese Cohort: Comparison With Classical Adenocarcinoma
Source: Front Oncol. 2020 Jun 9;10:917. doi: 10.3389/fonc.2020.00917 (PMC7296099; doi:10.3389/fonc.2020.00917)
Supplement: Supplementary file 2 [file Table_2.docx]

Supplementary Table 2. Clinicopathological features of AWMC and AC in different sites of left-sided colon

|  | Left-sided (Excluding rectal) | | |  | | Rectal | | | | |
| --- | --- | --- | --- | --- | --- | --- | --- | --- | --- | --- |
|  | AWMC (n=27) | | AC  (n=73)  n/% | |  | | | AWMC (n=30) | | AC  (n=85)  n/% |
|  | Without SRC  (n=23)  n/% | With SRC  (n=4)  n/% |  |  |  | | Without SRC  (n=27)  n/% | | With SRC  (n=3)  n/% |  |
| Sex |  |  |  | |  | | | |  |  |
| Male | 15 (65.2) | 2 (50.0) | 47 (64.4) | | 18 (66.7) | | | | 1 (33.3) | 49 (57.6) |
| Female | 8 (34.8) | 2 (50.0) | 26 (35.6) | | 9 (33.3) | | | | 2 (66.7) | 36 (42.4) |
| Age (yr), median | 57 (28-81) | 36 (28-68) | 60 (31-83) | | 57 (20-80) | | | | 44 (41-71) | 61 (32-91) |
| Tumor size (cm) |  |  |  | |  | | | |  |  |
| ≤5 | 11 (47.8) | 4 (100.0) | 38 (52) | | 16 (59.3) | | | | 3 (100.0) | 42 (49.4) |
| >5 | 12 (52.2) | 0 (0) | 15 (21) | | 11 (40.7) | | | | 0 (0) | 16 (18.8) |
| Unknown | 0 (0) | 0 (0) | 20 (27) | | 0 (0) | | | | 0 (0) | 27 (31.8) |
| T |  |  |  | |  | | | |  |  |
| Tis | 0 (0) | 0 (0) | 0 (0) | | 0 (0) | | | | 0 (0) | 0 (0) |
| T1 | 1 (4.3) | 1 (25.0) | 0 (0) | | 1 (3.7) | | | | 0 (0) | 2 (2.4) |
| T2 | 2 (8.7) | 0 (0) | 3 (4.1) | | 7 (25.9) | | | | 1 (33.3) | 8 (9.4) |
| T3 | 17 (73.9) | 3 (75.0) | 43 (58.9) | | 19 (70.4) | | | | 2 (66.7) | 46 (54.1) |
| T4 | 3 (13.1) | 0 (0) | 12 (16.4) | | 0 (0) | | | | 0 (0) | 9 (10.6) |
| Tx | 0 (0) | 0 (0) | 15 (20.6) | | 0 (0) | | | | 0 (0) | 20 (23.5) |
| N |  |  |  | |  | | | |  |  |
| N0 | 10 (43.5) | 0 (0) | 15 (20.5) | | 7 (25.9) | | | | 1 (33.3) | 21 (24.7) |
| N1 | 6 (26.1) | 0 (0) | 28 (38.5) | | 11 (40.8) | | | | 0 (0) | 23 (27.1) |
| N2 | 7 (30.4) | 3 (75.0) | 15 (20.5) | | 9 (33.3) | | | | 2 (66.7) | 18 (21.2) |
| Nx | 0 (0) | 1 (25.0) | 15 (20.5) | | 0 (0) | | | | 0 (0) | 23 (27.0) |
| M |  |  |  | |  | | | |  |  |
| M0 | 20 (87.0) | 3 (75.0) | 29 (39.7) | | 27 (100.0) | | | | 3 (100.0) | 39 (45.9) |
| M1 | 3 (13.0) | 1 (25.0) | 43 (58.9) | | 0 (0) | | | | 0 (0) | 42 (49.4) |
| Mx | 0 (0) | 0 (0) | 1 (1.4) | | 0 (0) | | | | 0 (0) | 4 (4.7) |
| AJCC Stage |  |  |  | |  | | | |  |  |
| I | 2 (8.7) | 0 (0) | 1 (1.4) | | 4 (14.8) | | | | 1 (33.3) | 4 (4.7) |
| II | 7 (30.4) | 0 (0) | 4 (5.5) | | 3 (11.1) | | | | 0 (0) | 10 (11.8) |
| III | 11 (47.8) | 2 (50.0) | 24 (32.8) | | 20 (74.1) | | | | 2 (66.7) | 25 (29.4) |
| IV | 3 (13.1) | 1 (25.0) | 43 (58.9) | | 0 (0) | | | | 0 (0) | 42 (49.4) |
| Unknown | 0 (0) | 1 (25.0) | 1 (1.4) | | 0 (0) | | | | 0 (0) | 4 (4.7) |
