# Supplementary material for: Clinicopathological Characteristics and Mutation Spectrum of Colorectal Adenocarcinoma With Mucinous Component in a Chinese Cohort: Comparison With Classical Adenocarcinoma
Source: Front Oncol. 2020 Jun 9;10:917. doi: 10.3389/fonc.2020.00917 (PMC7296099; doi:10.3389/fonc.2020.00917)
Supplement: Supplementary file 3 [file Table_3.DOCX]

Supplementary Table 3. Correlation of *KRAS* and *BRAF* status with clinicopathological features in AWMC without signet ring cell component

| Clinicopathological characteristics | *KRAS* status | | P | *BRAF* status | | P |
| --- | --- | --- | --- | --- | --- | --- |
|  | Wild (n=43)  n/% | Mutant  (n=47)  n/% |  | Wild (n=81)  n/% | Mutant  (n=9)  n/% |  |
| Sex |  |  | 0.456 |  |  | 0.302 |
| Male | 28 (65.1) | 27 (57.4) |  | 51 (63.0) | 4 (44.4) |  |
| Female | 15 (34.9) | 20 (42.6) |  | 30 (37.0) | 5 (55.6) |  |
| Age (yr), median | 64 (20-84) | 60 (28-81) | 0.555 | 61 (20-84) | 72 (40-79) | 0.232 |
| Tumor size (cm) |  |  | 0.799 |  |  | 0.497 |
| ≤5 | 19 (44.2) | 20 (42.6) |  | 34 (42.0) | 5 (55.6) |  |
| >5 | 23 (53.5) | 27 (57.4) |  | 46 (56.8) | 4 (44.4) |  |
| Unknown | 1 (2.3) | 0 (0) |  | 1 (1.2) | 0 (0) |  |
| Tumor location |  |  | 0.863 |  |  | 0.291 |
| Right-sided | 18 (41.9) | 21 (44.7) |  | 34 (42.0) | 5 (55.6) |  |
| Left-sided | 24 (55.8) | 26 (55.3) |  | 47 (58.0) | 3(33.3) |  |
| Multiple | 1 (2.3) | 0 (0) |  | 0 (0) | 1 (11.1) |  |
| T |  |  | 0.567 |  |  | 0.202 |
| Tis | 1 (2.3) | 0 (0) |  | 0 (0) | 1 (11.1) |  |
| T1 | 0 (0) | 2 (4.2) |  | 1 (1.2) | 1 (11.1) |  |
| T2 | 6 (14.0) | 6 (12.8) |  | 11 (13.6) | 1 (11.1) |  |
| T3 | 31 (72.1) | 31 (66.0) |  | 57 (70.4) | 5 (55.6) |  |
| T4 | 4 (9.3) | 8 (17.0) |  | 11 (13.6) | 1 (11.1) |  |
| Tx | 1 (2.3) | 0 (0) |  | 1 (1.2) | 0 (0) |  |
| N |  |  | 0.388 |  |  | 0.278 |
| N0 | 18 (41.9) | 21 (44.7) |  | 34 (42.0) | 5 (55.6) |  |
| N1 | 10 (23.3) | 19 (40.4) |  | 27 (33.3) | 2 (22.2) |  |
| N2 | 13 (30.2) | 7 (14.9) |  | 19 (23.5) | 1 (11.1) |  |
| Nx | 2 (4.6) | 0 (0) |  | 1 (1.2) | 1 (11.1) |  |
| M |  |  | 0.190 |  |  | 0.323 |
| M0 | 40 (93.0) | 41 (87.2) |  | 72 (88.9) | 9 (100.0) |  |
| M1 | 2 (4.7) | 6 (12.8) |  | 8 (9.9) | 0 (0) |  |
| Mx | 1 (2.3) | 0 (0) |  | 1 (1.2) | 0 (0) |  |
| AJCC Stage |  |  | 0.540 |  |  | 0.208 |
| I | 4 (9.3) | 5 (10.6) |  | 8 (9.9) | 1 (11.1) |  |
| II | 14 (32.6) | 14 (29.8) |  | 24 (29.6) | 4 (44.5) |  |
| III | 21 (48.8) | 22 (46.8) |  | 40 (49.4) | 3 (33.3) |  |
| IV | 2 (4.65) | 6 (12.8) |  | 8 (9.9) | 0 (0) |  |
| Unknown | 2 (4.65) | 0 (0) |  | 1 (1.2) | 1 (11.1) |  |
